# Anomalous widespread arid events in Asia over the past 550,000 years

**DOI:** 10.1093/pnasnexus/pgad175

**Published:** 2023-05-25

**Authors:** Igor Gustavo da Fonseca Carrasqueira, Luigi Jovane, André W Droxler, Carlos A Alvarez Zarikian, Luca Lanci, Montserrat Alonso-Garcia, Juan Carlos Laya, Dick Kroon

**Affiliations:** Departamento de Oceanografia Física, Instituto Oceanográfico da Universidade de São Paulo, Praça do Oceanográfico, 191, São Paulo, SP 05508-120, Brazil; Departamento de Oceanografia Física, Instituto Oceanográfico da Universidade de São Paulo, Praça do Oceanográfico, 191, São Paulo, SP 05508-120, Brazil; Department of Earth, Environmental and Planetary Sciences, Rice University, Houston, Texas 77005, USA; International Ocean Discovery Program, Texas A&M University, 1000 Discovery Drive, College Station, Texas 77845, USA; Department of Pure and Applied Science, University of Urbino, Via S. Chiara 27, 61029 Urbino, Italy; Geology Department, Universidad de Salamanca, Pza de Los Caídos S/n, 37008, Salamanca, Spain; Centro de Ciências Do Mar (CCMAR), Universidade Do Algarve, Campus de Gambelas, 8005-139, Faro, Portugal; Department of Geology and Geophysics, Texas A&M University, Mail Stop 3115, College Station, Texas 77843-3115, USA; Department of Geology and Geophysics, University of Edinburgh, Grant Institute, The King's Buildings, West Mains Road, Edinburgh EH9 3JW, UK

**Keywords:** Indian Summer Monsoon, millennial-scale events, arid conditions in Asia, paleoclimate, X-ray fluorescence

## Abstract

Records of element ratios obtained from the Maldives Inner Sea sediments provide a detailed view on how the Indian Monsoon System has varied at high-resolution time scales. Here, we present records from International Ocean Discovery Program (IODP) Site U1471 based on a refined chronology through the past 550,000 years. The record's high resolution and a proper approach to set the chronology allowed us to reconstruct changes in the Indian Monsoon System on a scale of anomalies and to verify their relationships with established records from the East Asian Monsoon System. On the basis of Fe/sum and Fe/Si records, it can be demonstrated that the Asia continental aridity tracks sea-level changes, while the intensity of winter monsoon winds responds to changes in Northern Hemisphere summer insolation. Furthermore, the anomalies of continental aridity and intensity of winter monsoon winds at millennial-scale events exhibit power in the precession band, nearly in antiphase with Northern Hemisphere summer insolation. These observations indicate that the insolation drove the anomalies in the Indian Summer Monsoon. The good correspondence between our record and the East Asian monsoon anomaly records suggests the occurrence of anomalous widespread arid events in Asia.

**Significance Statement**

The present work shows that Indian Monsoon winds respond mainly to changes in Northern Hemisphere insolation, while continental aridity in the Indian–Asia landmass follows glacial–interglacial cycles. These two components of the Indian Monsoon System, reconstructed from a single record, allowed establishing their lead–lag relationship without the recurrent problems of using different records with different chronologies. Furthermore, the present work shows that millennial-scale anomalous arid events have a good correspondence between our record of the Indian Monsoon System and the East Asian Monsoon System recorded in speleothems from China, demonstrating the occurrence of widespread anomalous arid events in the Asia.

## Introduction

The Indian Monsoon System (IMS) is characterized by a seasonal wind reversal driven by the seasonal cycle of solar heating over Asia in which dry northeasterly winds blow from the Indian–Asian landmass over the Arabian Sea during the boreal winter, while strong southwesterly winds transport heat and moisture across the Indian Ocean to the Indian subcontinent during the boreal summer (1–3). Indian Summer Monsoon (ISM) rainfall is one of the most intense recurring weather elements (4); their ability to transfer moisture to the Indian subcontinent and far away is of utmost importance to billions of people. After the International Ocean Discovery Program (IODP) Expedition 359 in 2015, great strides have been made toward understanding orbital control over the IMS (5–10). The sedimentary records, drilled in the Maldives Inner Sea, suggests that, in the last 500 kyr, the wind intensity and arid conditions in the dust source areas are related mainly to precession and eccentricity cycles (6, 7). These studies show the enormous potential of the Maldives dust record in the IMS research. However, to date, there is no publication about anomalies in the IMS on the Maldives records (11), although such occurring anomalous arid conditions were observed in a 640-kyr-long stalagmite δ^18^O record from Sanbao Cave situated in central China (12).

Here, we present high-resolution (1 cm) results from X-ray fluorescence (XRF) sediment core scanning along the upper 20 m below sea floor (mbsf) from IODP Site U1471 (Fig. S1), corresponding to the past ∼550 kyr. Site U1471 is located in a near central position in the Maldives Inner Sea on the most distal portion of a sediment drift extending from Goidhoo Atoll, one of the several atolls in the western chain of the Maldives Archipelago. This drill site provides the opportunity to record the ISM changes in a late Quaternary periplatform extended drift sediment sequence (details regarding the Site U1471 setting are available in Text S1 and Fig. S1). The site receives its terrigenous load, mainly during the winter, when a high-pressure cell with anticyclonic circulation develops over central India blowing cold and dry northeasterly winds (13), which carry dust from deserts in the Indian–Asian landmass to the eastern Arabian Sea (Fig. S2). Changes in the terrigenous content present in Maldives Inner Sea sediments have previously been linked to periods of expansion or retraction of arid zones in the Indian–Asian landmass (6, 14) (details regarding the supply of terrigenous sediments to the Maldives are available in Text S2 and Fig. S2).

Changes in Fe/sum and Fe/Si ratios are used as environmental indicators to assess aridity variability in the Indian–Asian landmass and changes in the Indian Winter Monsoon Winds (IWMW) intensity. Fe is an element with continental origin and is widely used to quantify the amount of lithogenic material in marine sediments. In the Maldives Inner Sea, a region far from river discharges, the Fe content is directly related to winter windblown dust, reflecting changes in aridity conditions in the Indian–Asian landmass (6). As a basis for our interpretation, the Fe/Ca ratio is a well-known indicator for terrigenous versus biogenic carbonate content (e.g. 15–17). We chose to normalize by dividing the sum of the elements to include carbonates from which calcium has been replaced by other elements (e.g. strontium).

The Fe/Si ratio is used here to represent the balance between clay-rich sediment (high Fe/Si ratio) and quartz-rich sediment (low Fe/Si ratio), commonly associated with the silt and sand fractions (18–20). We interpret changes in Fe/Si ratio as the effect of the sediment sorting during wind transport due changes in IWMW intensity. Fe and Si are immobile elements during Himalayan erosion and are thus not significantly affected by chemical weathering (21).

The anomaly on the East Asian Summer Monsoon was previously reported to occur in the rising branches of Northern Hemisphere summer insolation (NHSI) (12). To assess the ISM anomalies, we removed the influence of the main forcing founded in each of our records. The data were detrended by removing the influence of ice volume on our aridity record and the NHSI signal on our wind intensity record.

### Samples and results

The IODP Site U1471 (4°45.98′N and 73°08.11E; Fig. S1) is located at a water depth of 419.3 m in the Maldives Inner Sea, where the seafloor is essentially flat. The site comprises a continuous sedimentary record composed mainly by carbonate and very small amount of terrigenous material (22). The characteristics of this site allow to study the ISM through changes in terrigenous composition mainly consisting of aeolian dust.

The present work was carried out on 15 sedimentary core sections (each 1.5 m long) from holes U1471A, C, and D making up the uppermost 20 meters of the composite core section of Site U1471. Anhysteretic Remanent Magnetization (ARM@30mT) data are used to construct a single continuous record.

Confidence in XRF-scanning results is ensured by comparison between XRF core-scanning data, measured directly in the half-core surface (wet), with conventional XRF data obtained from dried and powdered samples (dry; Fig. S3A). High correlation coefficients were obtained for the elements Fe (*r* = 0.94431), K (*r* = 0.89381), and Si (*r* = 0.86234), and moderate correlation coefficients were obtained for Ti (*r* = 0.67319) and Al (*r* = 0.71857). This result supports the use of XRF-scanning data for elements Fe, K, and Si. High correlation coefficients between the two XRF methods were also obtained for the ln (Fe/Si) ratio (*r* = 0.73239; Fig. S3B) and for the Sr/sum data (*r* = 0.94054; Fig. S3C), and therefore, the data from XRF-scanning could be reliably used.

Our choice to use the Fe/sum record instead of Fe/Ca is ensured by the high correlation coefficient between Fe/Ca and Fe/sum (*r* = 0.98341) which shows that this choice does not affect our interpretation. For the use of Fe/Si as a record of IWMW, the Fe/sum and Si/sum records have a good correlation coefficient (*r* = 0.76217), with higher concentrations occurring during glacial periods, supporting the same aeolian origin.

### Chronology

Reconstructing the high-resolution chronology of sedimentary records from the Maldives Inner Sea has proved to be a difficult task. Although previous studies have found the same orbital periodicities in the records, they differ in the precise timing of events, which prevents the correlation with other records. At the nearby Site U1467, the chronology previously produced based on the correlation between the aridity record and the NHSI differs from the chronology produced based on records directly related to sea-level changes by up to 10 kyr (e.g. 6, 9).

To solve this problem, we tentatively constructed three different chronologies for Site U1471, following the methods previously used in other records from the Maldives Inner Sea (6–8, 23). The first age model (Fig. S4) was developed by comparing the Sr/sum curve with the global δ^18^O benthic foraminifera stack (24), the second age model (Fig. S5) was obtained by tunning the Fe/sum record filtered for the precession frequency band with the NHSI, and the third age model (Fig. S6) was obtained by linking the U1471 Sr/sum record, with the U1467 Sr/sum record (details regarding the three different chronologies are available in Materials and Methods; Text S3 and Figs. S4–S8).

The three age models produced for Site U1471 (the tie points obtained for the first, second, and third proposed chronologies are available in Tables S1–S3, respectively) differ in their chronology by up to ∼15 kyr in some intervals (Fig. S7), a difference also observed in the results obtained for Site U1467 (6, 9). This difference corresponds to about half precession cycle, therefore producing an inverse correlation between IWMW intensity [ln (Fe/Si)] and NHSI. Despite the differences, the three age models agree on the same chronology between 140 and 160 kyr (Fig. S7C; orange rectangle), allowing to match our aridity record (Fe/sum) and our record of IWMW intensity [ln (Fe/Si)] with the δ^18^O benthic foraminifera global stack (LR04) (24) and NHSI, respectively (Fig. S9). During this period, low (high) dust supply occurs during high (low) sea level (Fig. S9A), while weak (strong) winter winds occur during high (low) NHSI (Fig. S9B).

Cross-spectral analyses confirms this relationship for the whole record. The Fe/sum and LR04 records show high coherency in the eccentricity frequency for the three age models, while the ln (Fe/Si) record and NHSI show high coherency in the precession frequency for the first and third age models (Fig. S10). Surprisingly, the second age model provided the lower coherency between ln (Fe/Si) record and NHSI. We attribute this result to the forcings that act on the Fe/sum record. The Fe/sum record is driven firstly by changes in ice volume, which lags insolation, secondly by arid anomalies that increase wind-blown dust during the glacial terminations, and thirdly by the intensity of the winter winds that are in antiphase with the insolation. All these processes have components in the precession band, and the interplay between them generates a signal that does not necessarily correspond to the insolation and therefore produces an imprecise chronology.

Among the first and third proposed chronologies, we opted to use the first due to the high coherency between the Fe/sum record and LR04 in the obliquity frequency (Fig. S10). The obliquity largely controls the seasonal insolation contrast between the summer and winter hemispheres resulting in variations in cross-equatorial winds and moisture transport into the summer hemisphere (5, 25).

Based on this chronology, sedimentation rates at Site U1471 range from 1.57 to 7.87 cm per kyr (Fig. S8). The 1-cm spaced XRF measurements provide a temporal resolution range between ∼130 and 640 years, ideal for studying the monsoon anomaly.

### The continental aridity and wind intensity records

**Fig. 1.**
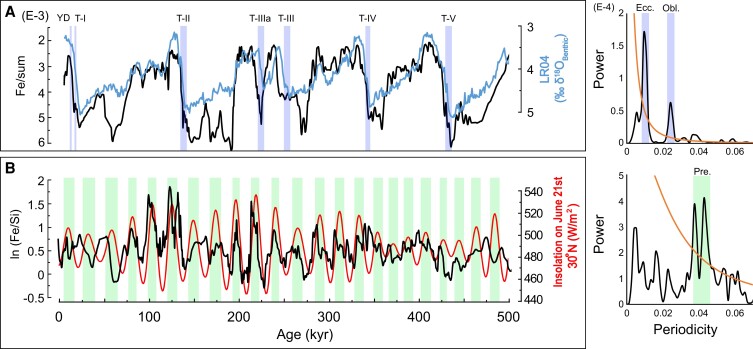
Continental aridity and wind intensity records. A) Records of continental aridity (Fe/sum) compared with LR04 and B) winter wind intensity (ln Fe/Si) compared with the insolation on June 21^st^.

Two main features characterize the IMS: wind reversal and continental rainfall. In relation to those both ISM characteristics, our records of continental aridity and winter wind intensity show dominant periodicities of glacial/interglacial cycles at the eccentricity and precession frequencies, respectively (Fig. 1). Different records distributed in this wide climatological system indicated that either one of these periodicities dominates the Asian Summer Monsoon (ASM) (e.g. 12, 26–28).

In a recent study (27), the ASM was discussed focusing mainly on speleothem records and climatological model simulations. The authors concluded that the ASM is mainly controlled by the NHSI with a phase lag of about 2 ky in relation to the insolation on June 21^st^ or almost in phase with July 21^st^ when the sea–land thermal gradient is maximum. However, some of the models used did not consider the global ice volume and the author also concluded that although atmospheric circulation has a well-known behavior, continental rainfall appears to be much more complex, with different responses in different parts of Asia. In contrast, in a marine core of Bay of Bengal, the dominance of 100-ka spectral power in precipitation proxies suggests sensitivity of South Asian Summer Monsoon precipitation to decreased ice volume and increased CO_2_ (28). Our record of continental aridity also suggests a large influence of global ice volume (Fig. 1A). Although part of this glacial–interglacial cyclicity may reflect changes in continental temperature, and other factors such as the extension and the type of vegetation cover rather than a pure ISM precipitation signal, changes in global temperature possibly forced changes in continental rainfall. Higher sea-surface temperatures in the Arabian Sea during interglacials (29, 30) may have led to increased moisture export. In addition, according to the Clausius–Clapeyron equation, for each 1°C of warming, saturated air contains 7% more water vapor, and even the extension and the type of vegetation cover could affect the continental rainfall through evapotranspiration that increases air humidity. Therefore, continental rainfall possibly was affected by glacial/interglacial cycles. Moreover, our record represents an average monsoon spread over a wide area, which minimizes the effects of local rainfall (27).

Unlike continental aridity, our record of winter wind intensity is mainly controlled by the inverse of NHSI with a phase lag of about 2 kyr from the insolation on June 21^st^ (Fig. 1B). This result shows a great similarity with the speleothem records in East Asia, raising a question: Could the wind intensity affect the cave’s δ^18^O record in eastern China? A recent study reported that δ^18^O speleothem records near the ASM moisture source have a wider range of glacial–interglacial variability, which then decreases landward (31).

### Anomalous arid events

**Fig. 2.**
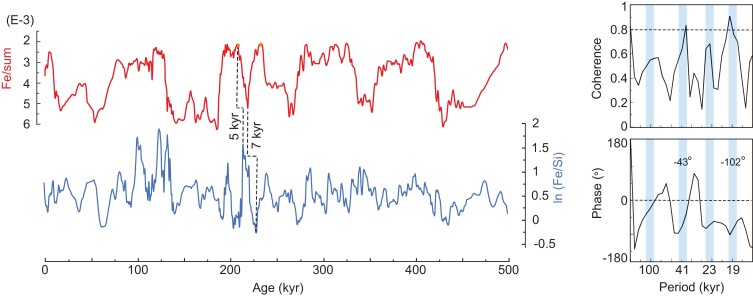
Phase relationship between records of continental aridity and wind intensity. Comparison between the Fe/sum and ln (Fe/Si) records. Note that at 214 and 228 kyr, the ln (Fe/Si) record precedes the Fe/sum record by around 5 and 7 kyr, respectively.

**Fig. 3.**
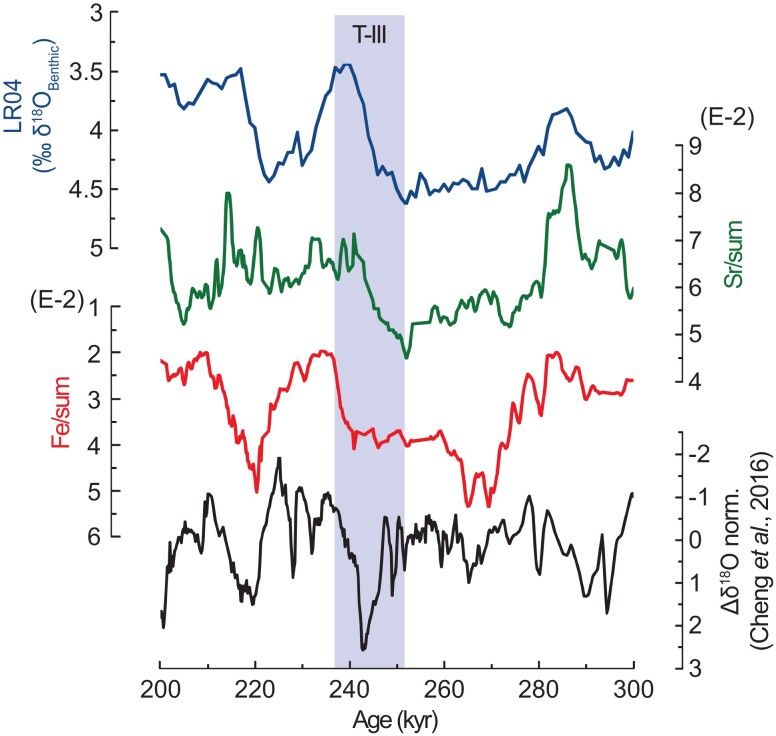
Widespread arid conditions in Asia during T-III. The Fe/sum record compared with Sr/sum record. The vertical bar indicates T-III. Note that during the termination, while the Sr/sum record tracks LR04 toward interglacial conditions, the Fe/sum data remains high, indicating continuing glacial arid conditions. In the same period, a weak east Asia monsoon interval was previously reported (12).

The comparison between Fe/sum and ln (Fe/Si) records shows significant coherency for precession and obliquity, with a phase lag of 102° (∼5.4 kyr) and 43° (∼4.9 kyr), respectively, indicating that changes in IWMW intensity precede changes in continental aridity (Fig. 2). This is clearly visible at 214 and 228 kyr, when the ln (Fe/Si) record precedes the Fe/sum record by around 5 and 7 kyr, respectively (Fig. 2). A possible explanation is that the ln (Fe/Si) record responds directly to changes in NHSI, while the Fe/sum record tracks sea-level changes and therefore incorporates the delay associated with changes in global ice volume. However, during glacial termination three (T-III), the Fe/sum record lags the Sr/sum and the sea level by ∼10 kyr (Fig. 3). This result suggests the maintenance of arid conditions in the Indian–Asian landmass during the MIS 8/7 transition (between 252 and 240 kyr). Surprisingly, this arid period coincides with anomalously Weak East Asian Monsoon Interval (WEAMI) (12). The occurrence of extended aridity in our record concomitant with WEAMI indicates that the T-III was characterized by widespread arid conditions in Asia (Fig. 3).

### Anomalous widespread arid events in Asia

WEAMIs were previously reported during the last seven glacial terminations, during rising NHSI (12). To verify the occurrence of anomalous widespread arid events in Asia and its correlation with winter wind intensities, we removed the effect of global ice volume from our aridity record (Fe/sum), producing a new record of aridity anomaly (Δ Fe/sum norm.), and we also removed the effect of NHSI from our IWMW intensity record [ln (Fe/Si)], producing a new record of IWMW anomaly [Δ ln (Fe/Si) norm.]. We arbitrarily used the insolation on June 21^st^ since our chronology was linked to the LR04 curve, and the authors (24) used a simple ice model based on June 21^st^ insolation.

**Fig. 4.**
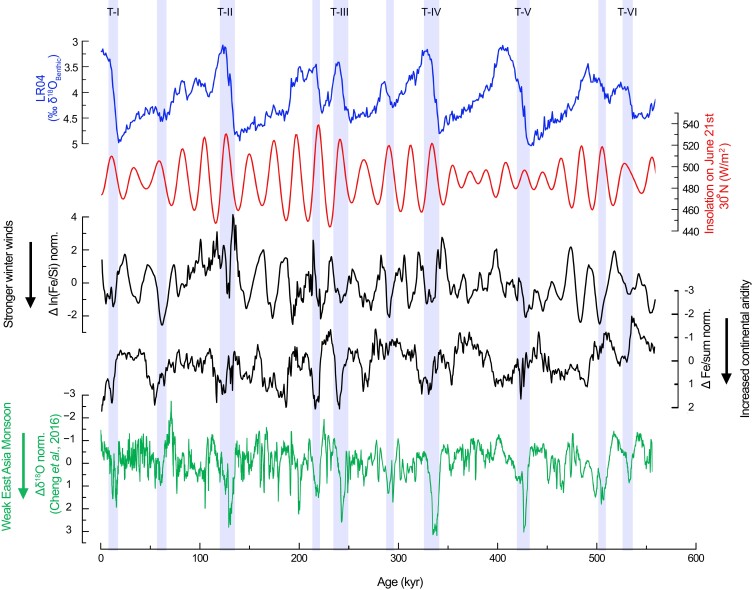
Comparison of Asian monsoon anomaly records with the global ice volume and the NHSI. Aridity anomaly (Δ Fe/norm sum.) and winter wind anomaly [Δ ln (Fe/Si) norm.] in the Indian–Asian landmass in comparison with anomaly in the EASM (12) (Δ δ18O norm.). The increase in arid anomaly conditions occurred in both the EASM and the Indian–Asian landmass during the increase in insolation. Note that the main aridity anomaly events occurred concurrently with the major drops in the values of LR04 during sea-level rise.

**Fig. 5.**
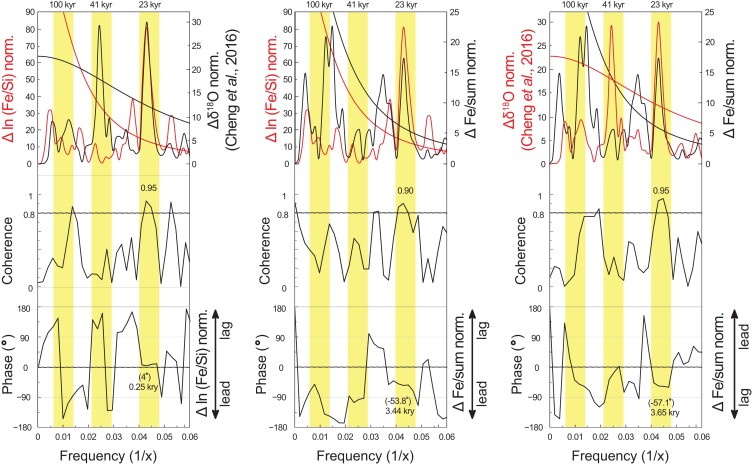
Cross-spectral analyses between anomaly records. Cross-spectral analyses between records of the aridity anomaly (Δ Fe/sum), IWMW anomaly [Δ ln (Fe/Si)], and the anomaly in the EASM.

The results (Fig. 4) show the occurrence of increased aridity anomaly with stronger winter wind intensities through the Maldives dust record occurring together with major WEAMIs revealed by the Sanbao Cave record (12). Cross-spectral analyses show high coherence in the precessional frequency between these records (Fig. 5). The results indicate that the IWMW anomaly is in phase with the East Asian Monsoon anomaly (Fig. 5).

**Fig. 6.**
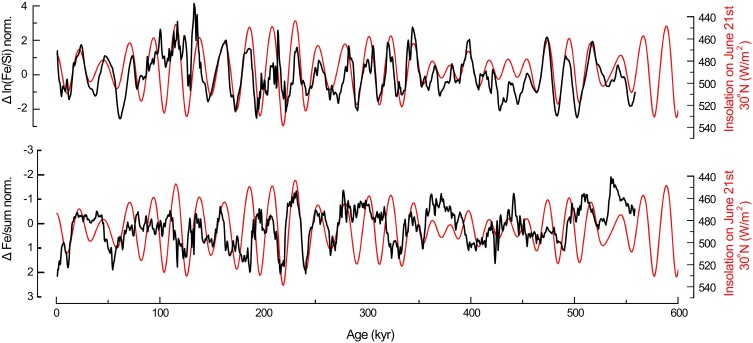
Anomaly records in the context of insolation. Aridity anomaly (Δ Fe/sum) and IWMW anomaly [Δ ln (Fe/Si)] compared with inverse NHSI.

Previous studies suggest that during rising insolation, the decay of the Northern Hemisphere ice sheets resulted in the flux of ice and melt water into the North Atlantic, which slowed down the Atlantic meridional overturning circulation, generating a cold anomaly over the North Atlantic that resulted in WEAMIs through atmospheric teleconnection (32, 33). This process generates an anomaly in the EASM close to antiphase with NHSI (12). Our record of the IWMW anomaly behaves close to antiphase with NHSI (Fig. 6), as well as the EASM anomaly (12).

Comparison between spectra of the aridity anomaly record in the Indian–Asian landmass (Δ Fe/sum) and spectra of the IWMW anomaly record [Δ ln (Fe/Si) norm.] shows dominance of precession (Fig. 5), which indicates the same climatic mechanism (e.g. decay of ice sheets during rise NHSI) driven by the insolation, acting on the anomalies.

The close correlation between the IWMW anomaly and the inverse (∼180o) of NHSI, with no power in ∼100 kyr period, indicates that the anomaly acts, dampening the effect of insolation on IWMW. Could these anomalies dampen the direct effect of insolation on the ISM to the point of allowing a greater control by the ice volume at high latitudes over the monsoons at low latitudes?

The wind intensity strongly depends on thermal gradient between the continent and the Indian Ocean. Our record of IWMW is strongly controlled by the NHSI, which suggests that periods of glacial maxima are not defined by changes in sea–land thermal gradient and, therefore, cold conditions may have developed both in the Indian subcontinent and in the Indian Ocean.

The wind system exerts an important control over the continental rainfall but is not the only one forcing. Surface ocean temperature, air temperature, snow cover, and continental vegetation cover affect the water cycle (34–36). Compared with a glacial period, during an interglacial, a warmer sea surface could export more humidity to the air, which in turn, due to is higher temperatures, incorporates more moisture until it saturates and thus transports more humidity to the continent. On the continent, warmer temperatures reduce the snow cover and could lead to an expanded and denser vegetation cover with larger species increasing evapotranspiration which in turn could led to increased rainfall.

## Materials and methods

### Anhysteretic remanent magnetization

ARM experiments were made over U-channels collected from 15 split half-core sections (each of 1.5 m long). The experiments were performed on the Institute of Astronomy and Geophysics from University of São Paulo using the triaxial cryogenic magnetometer (2G, mod. 755). The protocol consisted in exposing the samples to an alternating flow field of 30 mT that falls gradually to zero in the presence of a 50 microtesla (μT) DC bias field (37). The ARM@30mT is oriented in the direction of the applied direct field and indicates the presence of fine-grained magnetite (38, 39).

### XRF

XRF-scanning measurements were taken at 1-cm intervals directly on the surface of the split half-core section (each of 1.5 m long) using a third-generation XRF-Scanning Avaatech at the IODP’s Gulf Coast Repository, at Texas A&M University. The XRF-Scanning X-ray source was an Oxford Instruments 100 W Neptune X-ray tube with a rhodium (Rh) target, and data were acquired using a Canberra X-PIPS silicon drift detector, with 150-eV X-ray resolution at 5.9 keV and a Canberra Digital Spectrum Analyzer model DAS 1000. The surface of the half-core at room temperature was covered with a 4-μm-thick Ultralene plastic film to avoid contamination and desiccation of the sediment. Due to the physical configuration of the XRF-scanner, the first and last 4 cm of each section were not measured. The measurements were performed at 1-cm resolution, with a beam size of 1 × 1 cm for two consecutive runs of the same section, the first one at 9 kV, 0.25 mA, and 6 s and the second one at 30 kV, 1.25 mA, and 6 s. The first scan provided data for the elements Mg, Al, Ba, Ca, Cl, Cr, Fe, K, Mn, P, S, SI, Rh, and Ti and the second for Bi, Cu, Br, Fe, Ga Nb, Ni, Mo, Pb, Rb, Sr, Y, Zn, and Zr. The XRF scanner measures the chemical composition of the sediment, as intensity of elements in counts per second (cps), which are proportional to chemical concentrations.

### XRF-scanning confidence

Confidence in XRF-scanning data is affected by specimen inhomogeneity, variable water content, change in grain size, and other unknown factors that change the element intensities (40–42). To improve confidence in XRF-scanning data, discrete measurements on conventional XRF were performed on 42 samples collected at each 3-cm intervals from core section U1471C-1H-3W (3 to 4.5 mbsf). The samples, taken in 5-g aliquots of core material, were dried and powdered in an agate mortar. Powdered samples were measured in the conventional XRF Rigaku Supermini200 at University of São Paulo. Correlation coefficient between the two methods was used to determine the reliability of the XRF-scanning data.

### Element normalization

In the results presented here, the raw data of elements of interest in each sample were normalized by dividing the sum of all elements detected in the respective sample (element/sum). This procedure (called closure) removes the main problems related to unprocessed sample (e.g. specimen inhomogeneity, variable water content, and change in grain size) measured by the XRF-scan method and matrix effect that also may affect the data, scattering, absorbing, and enhancing an element intensity by the presence of the other elements (40–42). It allows the measurements to be expressed in proportions, where the sum of all elements measured in a sample comprises the unit (42).

Bulk elemental concentration may be subjected to large fluctuations caused by variable proportions of biogenic versus detrital minerals. To evaluate the changes in detrital elements as paleoenvironmental proxies, normalization by Al and Ti, which are the most refractory elements (43), has been widely used (44, 45). However, the use of Al and Ti is unreliable due to the low correlation coefficient found between the conventional XRF and the XRF-scanning. Alternatively, we opted to use the Fe/Si ratio. This choice was supported by the high correlation coefficient (*r* = 0.73239) between the ln (Fe/Si) ratio data from two methods (Fig. S3B).

The element ratios related to the paleoenvironmental indicators used in this work are plotted in the logarithmic (ln) form. Since they are nonsymmetric (e.g. between Fe/Si and Si/Fe), and there is no rule to suggest which element should be the numerator or the denominator, this procedure put symmetry in an element ratio with an unbiased choice. The relation between weight and proportions of the measured intensities presented as log ratio is also expected to be linear (42).

### Age model

The first age model (Fig. S4) was obtained by comparing the Sr/sum curve with δ^18^O benthic foraminifera stack (24). Sea level is the fundamental driving force behind flooding and exposure of the shallow-water production areas and thus fine bank-derived carbonate production and export. According to Boardman et al. (46) and Droxler et al. (47), during sea-level highstands, when carbonate platform flat tops are flooded, the Sr-rich aragonite production and export reach their maxima. This production falls during lowstands, when platform tops are exposed and karstified, restricting production to terraces. In sediments from Maldives Inner Sea, Paul et al. (23) reported a very strong correlation between the aragonite record and the fine grain-size fraction. According to the authors, the sediment redistribution through ocean currents does not seem to influence the glacial/interglacial variations in aragonite and the fine grain-size fraction significantly. Furthermore, the authors suggest that the aragonite content can be used to better quantify the timing and extent of changes in the Pleistocene sea level. Aragonite preservation in U1471 record is considered good in the studied interval, as supported by XRD data that presented high aragonite concentration and zero dolomite (22) and the common occurrence of fossil pteropods in the late Pleistocene sediments at U1471 (direct observation) and at U1467 (48).

A second age model (Fig. S5) was obtained assuming that lithogenic input in the Maldives Inner Sea is directly related to aridity in the source area and responds to changes in NHSI. During periods of low NHSI, the arid zone expansion or strengthened IWMW promoted an increased Fe-rich dust supply. This assumption follows previous studies that reported the appearance of precession cycles in the lithogenic content of sediments from the Maldives Inner Sea (6, 7, 14). To create this second age model, we applied the chronology obtained with the first age model in U1471 Fe/sum record. Then, the record was linearly interpolated for 1 kyr and filtered using a band-pass finite impulse response (FIR) filter in the software PAST (49) for frequencies between 0.06 (1 kyr/0.06 = 16.67 kyr) and 0.035 (1 kyr/0.035 = 28.57 kyr), surrounding the precession frequency band. The filtered high (low) values were tuned in zero lag phase with the low (high) values of the June 21^st^ insolation at 30°N (50).

The third age model (Fig. S6) was obtained by linking the U1471 Sr/sum record, with the U1467 Sr/sum record. Site U1467 (51) is situated 16.4 km northeast of Site U1471 near the center of the Inner Sea (Fig. S1) and has its chronology established by the correlation of the U1467 δ^18^O *Globigerinoides ruber* white record (9) with δ^18^O benthic foraminifera global stack (24) (LR04). The correlation between these two sites is supported by the fact that sediment redistribution in Maldives Inner Sea seems to be not affected by ocean currents (23).

### Normalization and detrending

To remove the global ice volume component (LR04) (24) from our aridity record (Fe/sum) and to remove the NHSI (50) (June 21^st^ insolation at 30˚N) from our intensity of IWMW record [ln (Fe/Si)], we first normalized each data set so the mean equals zero and standard deviation equals one (that is, zero–mean normalization). Data were linearly interpolated to a resolution of 500 years allowing point-to-point alignment. After standardization and equal spacing to the 500-year interval, the detrended Fe/sum data (Δ Fe/sum norm.) was obtained by subtracting LR04 from the Fe/sum record and the detrended ln (Fe/Si) data [Δ ln (Fe/Si) norm.] was obtained by subtracting NHSI from the ln (Fe/Si) record.

### Spectral density and cross spectral analysis

To perform spectral analysis, all data were linearly interpolated for a 1 kyr resolution. Spectral density was assessed using the Multitaper method (52). Cross-spectral analyses (53) were made using Acycle software (54). The analysis provides the coherence and the phase between two records, spanning 257 frequency bands ranging from 0 to the Nyquist frequency. Coherency is a measure of the maximum linear correlation between two signals, and ranges from 0 to 1 with significance values assessed at 0.8. Phase estimates span −180° to 180° and can be directly converted to time for specific frequency band (e.g. a phase lag of 90° at the precession band indicates a lag of 90°/360° × 23 kyr = 5.75 ky).

## Supplementary Material

pgad175_Supplementary_Data

## Data Availability

The ARM@30mT, Fe/sum, ln (Fe/Si), (Δ Fe/sum norm.), and [Δ ln (Fe/Si) norm.] data are freely available from https://doi.org/10.6084/m9.figshare.21545439 (Dataset 55).
