# Supplementary material for: Anomalous widespread arid events in Asia over the past 550,000 years
Source: PNAS Nexus. 2023 May 25;2(6):pgad175. doi: 10.1093/pnasnexus/pgad175 (PMC10244003; doi:10.1093/pnasnexus/pgad175)
Supplement: pgad175_Supplementary_Data [file pgad175_supplementary_data.pdf]

Supplementary material for

Anomalous widespread arid events in Asia over the past 550,000 years

Igor Gustavo da Fonseca Carrasqueira[1], Luigi Jovane[1], André W. Droxler[2], Carlos A. Alvarez Zarikian[3], Luca Lanci[4], Montserrat Alonso-Garcia[5], Juan Carlos Laya[6], Dirk Kroon[7†]
[1]Oceanographic Institute of the University of São Paulo, São Paulo, Brazil
[2]Department of Earth, Environmental and Planetary Sciences of Rice University, Texas, United States
[3]International Ocean Discovery Program, Texas A&M University, Discovery Drive, College Station, TX 77845, USA
[4]Università degli Studi di Urbino, Urbino, Italy
[5]Universidad de Salamanca, Geology Department, Pza de los Caídos s/n, 37008 Salamanca, Spain & Instituto Português do Mar e da Atmosfera (IPMA), Av. Alfredo Magalhães Ramalho 6, 1495-006 Lisboa, Portugal
[6]Department of Geology and Geophysics, Texas A&M University, College Station, TX 77843-3115
[7]School of GeoSciences, University of Edinburgh, Grant Institute, The King's Buildings, West Mains Road, Edinburgh EH9 3JW, United Kingdom.
† Deceased.

Supplementary texts

Text S1. The Maldives Archipelago is an isolated tropical carbonate platform in the central equatorial Indian Ocean, located southwest from India (fig. S1). In the central part of the Archipelago, the north/south-oriented dual row of atolls enclose the Maldives Inner Sea, an internal basin with water depths of up to 550 meters. The sediments accumulating in the Inner Sea are referred to as periplatform ooze, consisting of a mixture of mostly fine particles exported from the atolls made of aragonite and magnesian calcite, and pelagic components settling down from the upper part of the water column, planktic foraminifers and coccoliths made of calcite and pteropod tests made of aragonite (56-58), locally accumulated in drift prograding sediments bodies (59).

The uppermost 20 meters below the seafloor (mbsf) are dominated by pelagic components, with small neritic and terrestrial contribution. The lithology is characterized by unlithified well-preserved planktonic foraminifer-rich, fine- to medium-grained packstone to wackestone (57). Components include abundant planktonic foraminifers and well-preserved benthic foraminifers, ostracods, echinoderm spines, pteropods, sponge spicules, calcareous nannofossils (e.g., *Gephyrocapsa* sp.), and mollusk fragments (57). X-Ray Diffraction (XRD) measurements in this interval made by (57) presented aragonite concentration reaching around 40 % and zero dolomite.

Text S2. To assess the flow of dust to the Maldives archipelago we used satellite data. Aerosol Optical Thickness and Aerosol Ångström Exponent are important parameters in understanding the status of ambient aerosol concentration. Aerosol Optical Thickness analyses are based on the fact that the particles change the way the atmosphere reflects and absorbs visible and infrared light. An Aerosol Optical Thickness of less than 0.1 indicates a crystal clear sky with maximum visibility, whereas a value of 1 indicates very hazy conditions (60). The Aerosol Ångström Exponent is basically a quantitative indicator and inversely proportional to the size of the aerosol particles. When the Aerosol Ångström Exponent is close to 1.0 is considered to be black carbon-rich aerosol from fossil fuel burning, and when it is higher than 1, it is often considered to be an indication of the presence of dust (61-64).

During boreal summer, strong southwesterly winds together with northerly Shamal winds deliver a huge amount of aeolian dust sourced in the Arabian Peninsula and Nubian Desert into northwestern Arabian Sea (65-67) (fig. S2A). Satellite compiled data (2002-2020) from seasonal distribution of Aerosol Optical Thickness, show an increase in the flux of aerosol in the northwestern Arabian Sea during Summer Monsoon (68) (fig. S2B); although only a small portion

of these aeolian dust reach the eastern Arabian Sea where the Maldives Archipelago is located (69, 70).

In the winter, a high-pressure cell with anticyclonic circulation develops over central India blowing cold and dry northeasterly winds across the Indian east coast with a limited sea travel (71) (fig. S2A). These northeasterly winds carry dust from deserts in the Indian-Asian landmass to the eastern Arabian Sea. Satellite compiled data (2002-2020) from seasonal distribution of Aerosol Ångström Exponent, show an increase in the flux of fine dust particles (high values) toward the Maldives Archipelago during the winter monsoon (68) (fig. S2E), clearly distinct from the input of coarse sediments (low values) during the summer (68) (fig. S2D).

Changes in the lithogenic fraction of sediments from Maldives Inner Sea was previously associated with changes in the amount of aeolian dust transported from the Indian-Asian landmass during the winter monsoon (69,72). This result was explained by the dominance of fine particles in the lithogenic fraction (70). Changes in continental aridity recognized in bulk sediments from the Maldives Inner Sea through the Fe/K record are associated with changes in global ice volume (69). On the other hand, the summer wind intensity, recognized in the coarse grain-size fraction, presented higher frequency orbital-driven cycles in the precessional (23 kyr) and the obliquity (41 kyr) bands (70).

Text S3. The 1st age model for the Site U1471 provided 19 tie points (table S1) allowing to recognize all the marine isotope stages from MIS 1–14 (fig. S4). Sedimentation rates range from 1.6 cm/kyr to 7.9 cm/kyr, with an average of 3.7 cm/kyr. High sedimentation rates occurred at glacial to interglacial transitions, during increasing orbital eccentricity, except for the MIS 7, when high sedimentation rates occurred during orbital eccentricity fall. The highest values occur during MIS 8/7 and MIS 6/5 transitions.

The 2nd age model provided 52 tie points (table S2). Sedimentation rates range from 0.8 cm/kyr to 9.2 cm/kyr, with an average of 3.6 cm/kyr (fig. S5).

The 3rd age model provided 21 tie points (table S3) allowing to recognize all the marine isotope stages, from MIS 1–14 (fig. S6). Sedimentation rates range from 2.24 cm/kyr to 8.25 cm/kyr, with an average of 3.7 cm/kyr. High sedimentation rates occurred during high sea level, in periods of high orbital eccentricity during MIS 7 and 5.

The three obtained age models present close chronologies over the glacial/interglacial periods, although, in some points, the ages differ by up to 20 kyr (fig. S7). In the period between 140 to 160 kyr the three obtained age models agree with a same chronology (fig. S7C; orange rectangle).

Sedimentation rates for the three age models agree with low (high) sediment accumulation during glacial (interglacial) periods. Moreover, especially high sediment accumulation rates occurred during MIS 5 and 7, during periods of high orbital eccentricity (fig. S8).

Supplementary figures

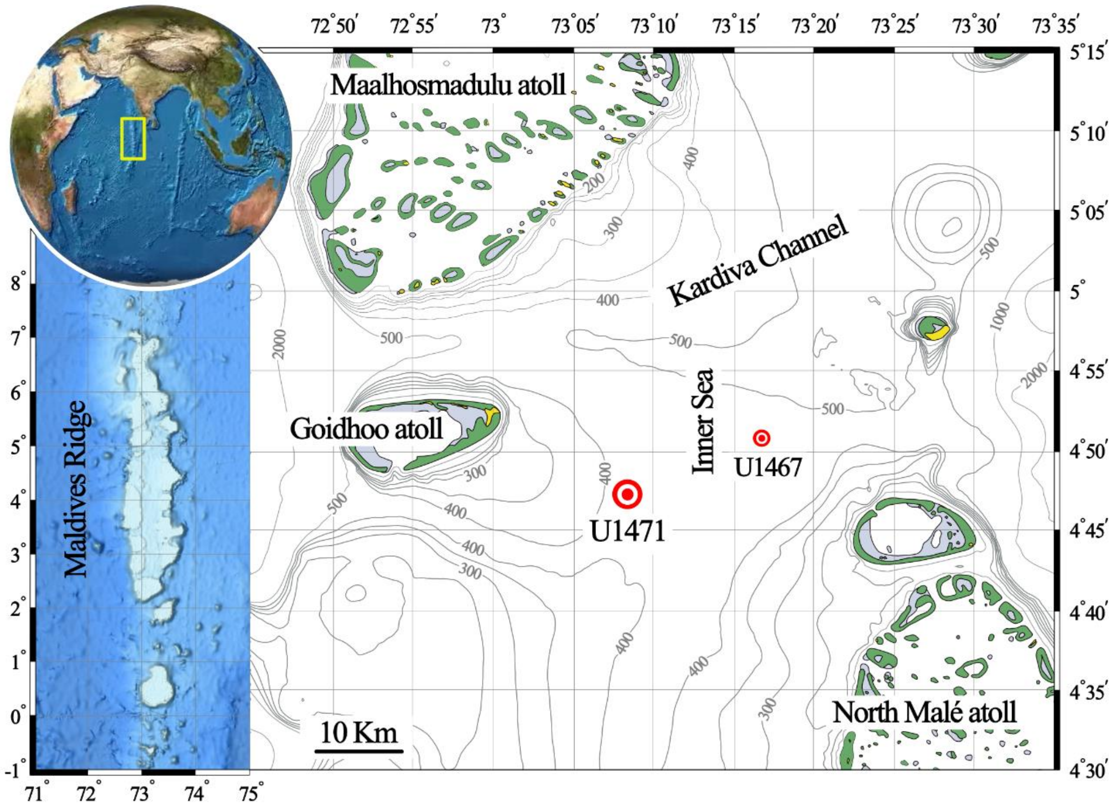

Figure S1. Location of the Maldives Archipelago (yellow rectangle) along the central part of the Chagos-Laccadives Ridge in the Indian Ocean. The Site U1471 is situated in the deepest part of the Inner Sea (adapted from 57).

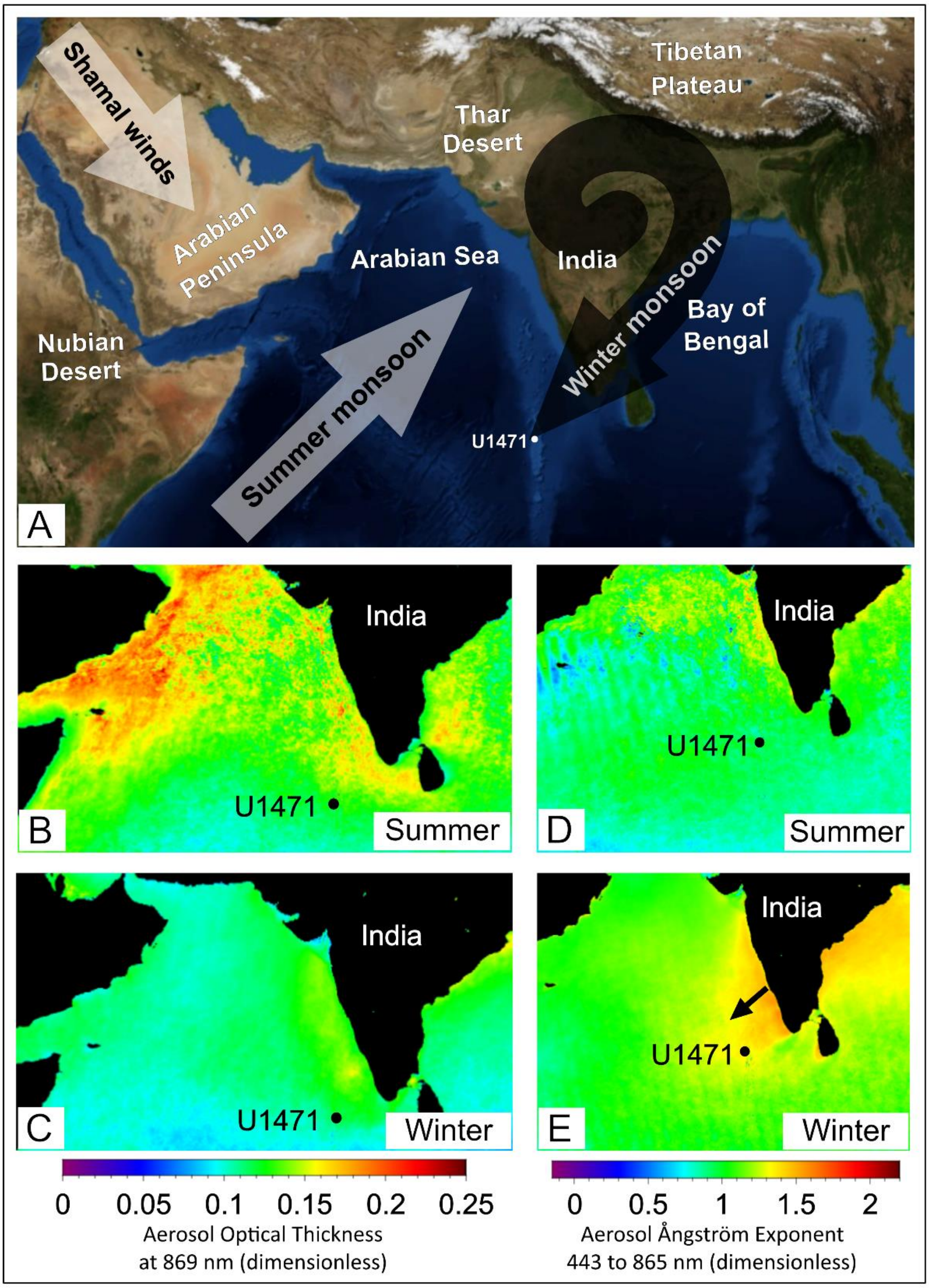

Figure S2. (A) Satellite image of the IMS domain with the monsoon wind scheme during the summer (white arrows), and during the winter (black arrows). The figure also shows satellite compiled data (2002-2020) from (B) summer and (C) winter distribution of the Aerosol optical thickness at 869 nm and (D) summer and (E) winter distribution of the Aerosol Ångström Exponent 443 to 865 nm.

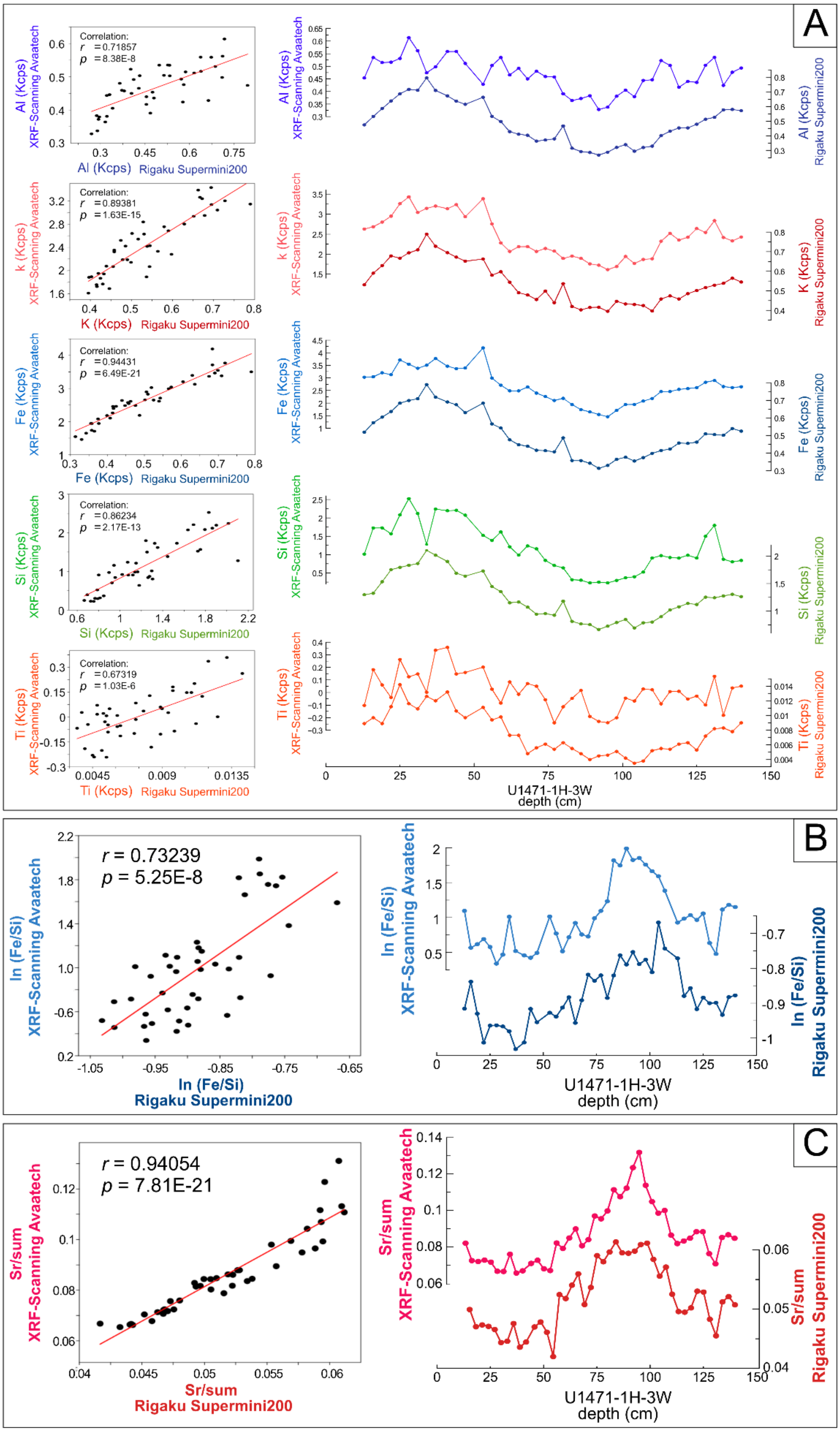

Figure S3. Comparison between the data obtained by conventional XRF Rigaku Supermini200 on 41 discrete samples collected from core section U1471-1H-3W with the values obtained with Avaatech XRF core-scanner at their respective depths, is shown **(A)** for Al, K, Fe, Si and Ti, (B) for the Fe/Si ratio, and (C) for the Sr/sum.

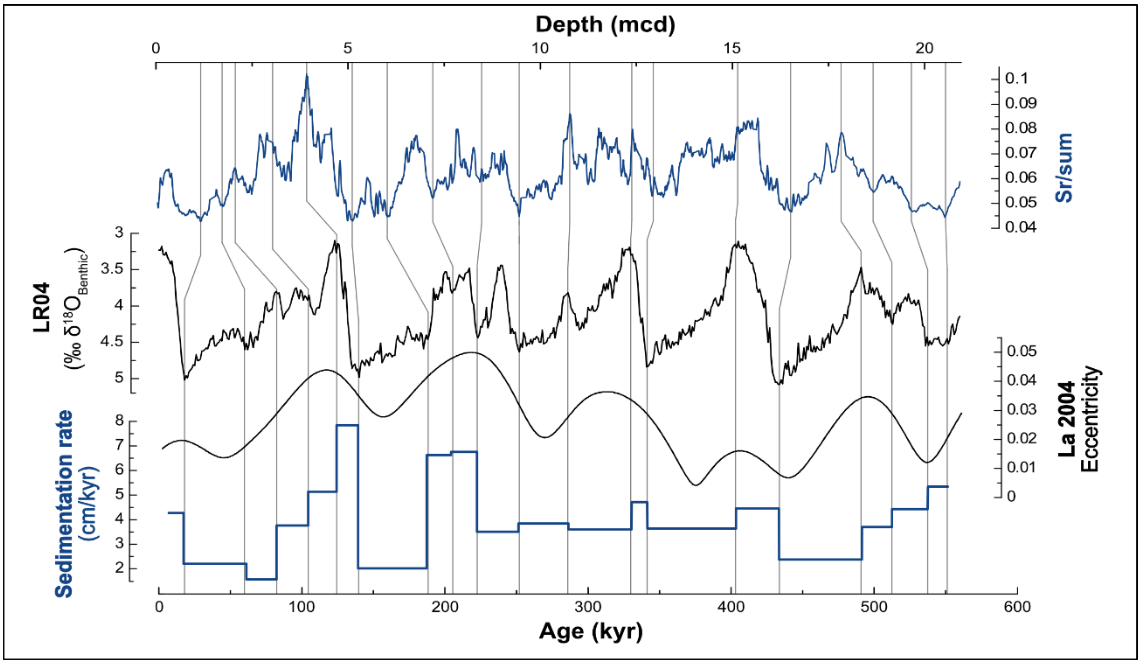

Figure S4. The 1st proposed age model for the Site U1471; vertical lines show the obtained tie points. From the top: correlation between the Sr/sum record, global $\delta^{18}O$ benthic foraminifera stack (73), orbital eccentricity (74) and sedimentation rates.

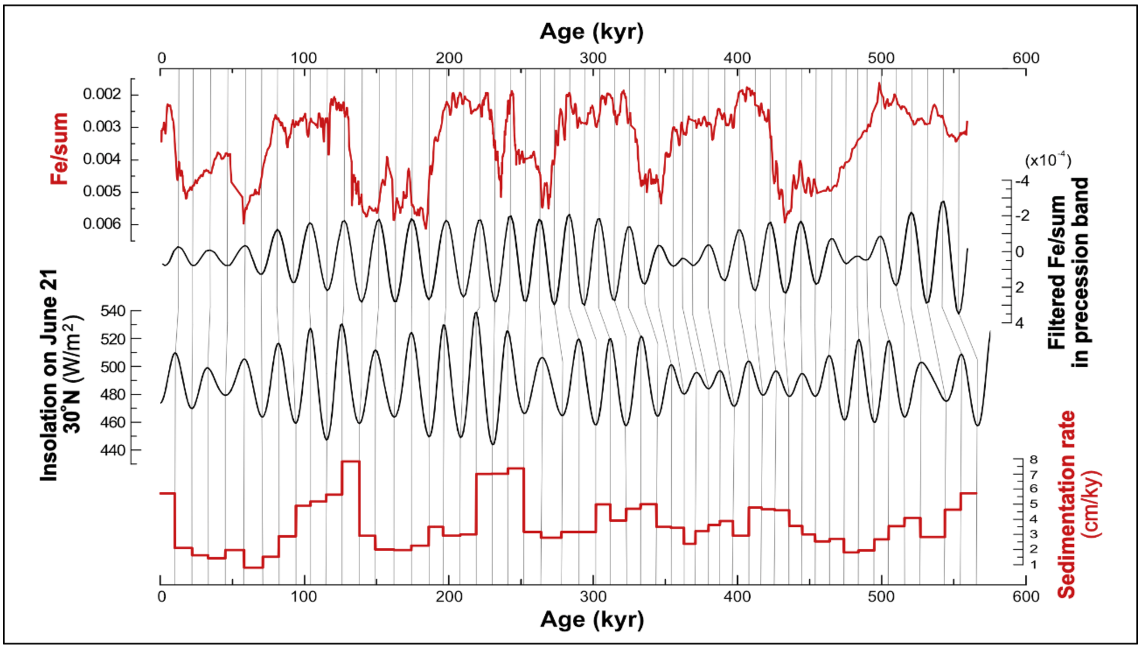

Figure S5. The 2nd proposed age model for the Site U1471; vertical lines show the obtained tie points. From the top: Fe/sum and filtered Fe/sum correlated with June 21st insolation at 30˚N (74), and sedimentation rates.

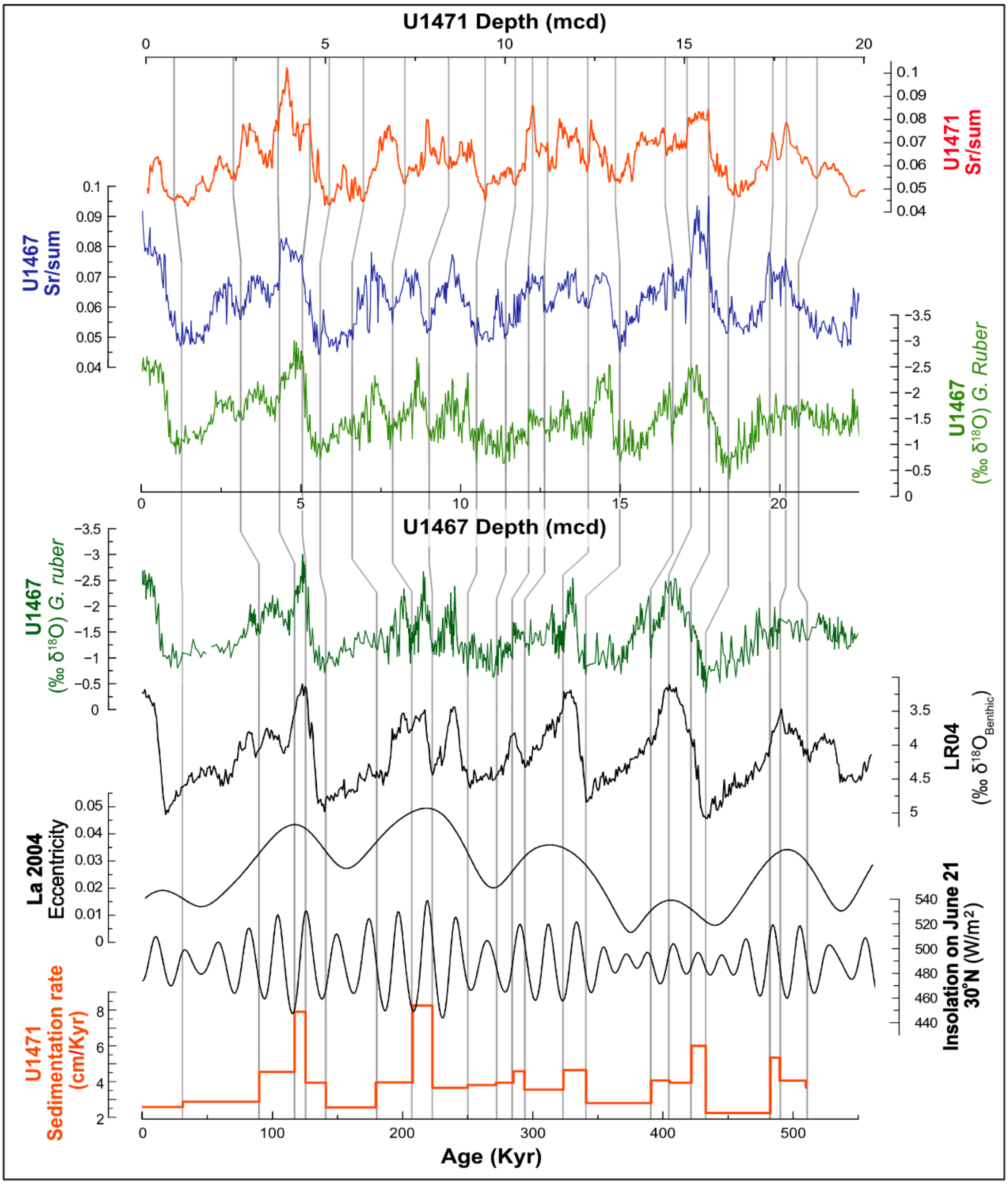

Figure S6. The 3rd proposed age model for the Site U1471; vertical lines show the obtained tie points through correlation with well-dated Site U1467. From the top: the U1471 Sr/sum, U1467 Sr/sum, U1467 $\delta^{18}O$ *Globigerinoides ruber* white records versus depth and age (75), the global $\delta^{18}O$ benthic foraminifera stack (73) (LR04), orbital eccentricity (La 2004) and NHSI (74) (June 21st insolation at 30˚N) and sedimentation rates.

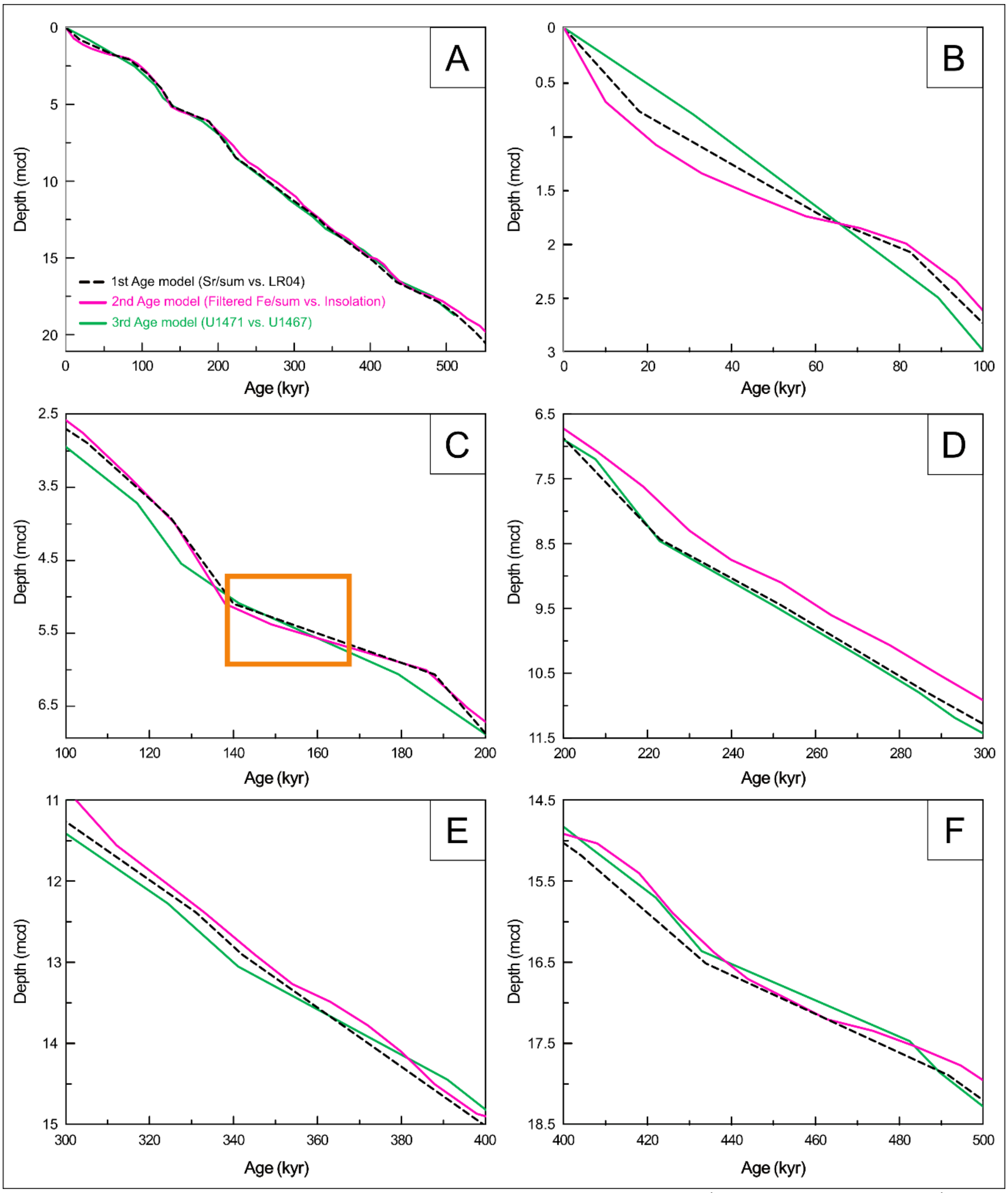

Figure S7. Comparison between the 1st (black dashed line), the 2nd (pink line) and the 3rd (green line) obtained chronologies. The six panels show comparisons for different time intervals: **(A)** from 0 to 500 kyr, (B) from 0 to 100 kyr, (C) from 100 to 200 kyr, (D) from 200 to 300 kyr, (E) from 300 to 400 kyr, and (F) from 400 to 500 kyr. Note that during the period between 140 and 160 kyr the three age models obtained agree with the same chronology (orange rectangle; C).

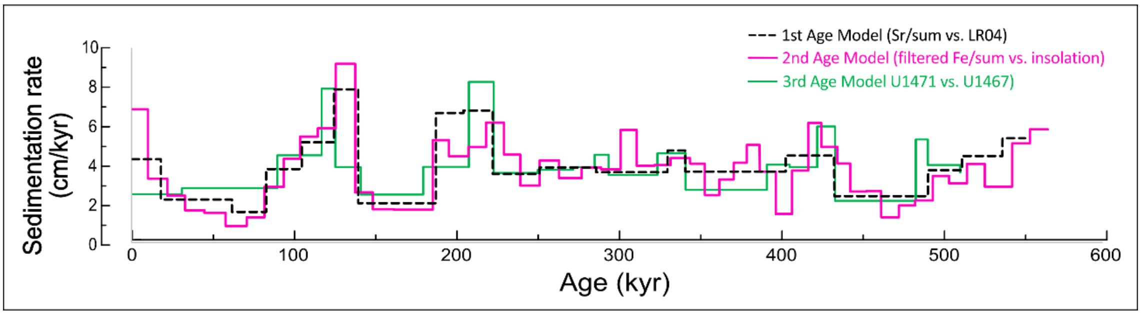

Figure S8. Comparison between the sedimentation rates obtained with the three age models. Sedimentation rates for the 1st, 2nd and 3rd proposed age models are shown in black, pink and green respectively.

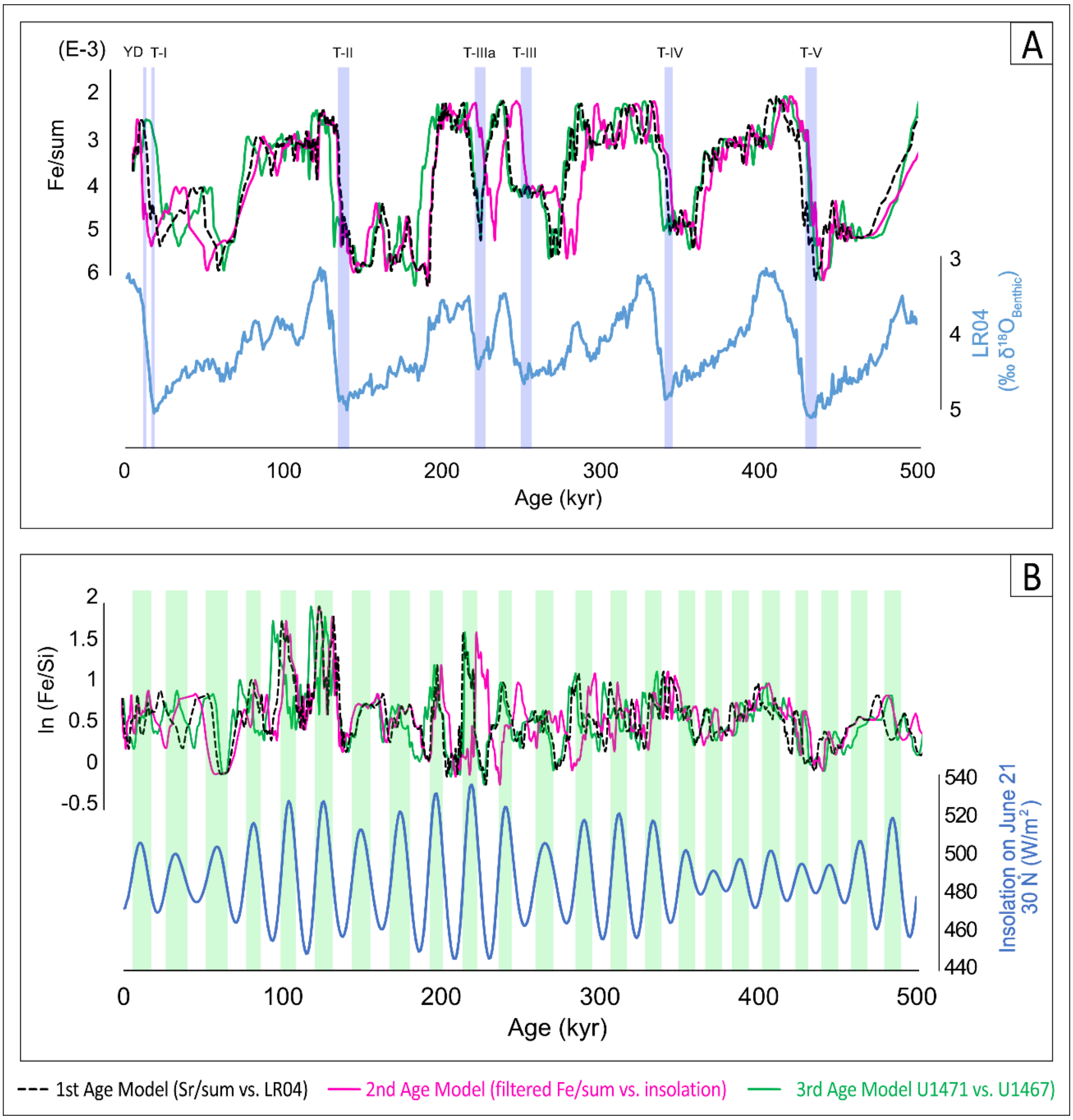

Figure S9. (A) Fe/sum record compared to $\delta^{18}O$ benthic foraminifera stack (73) (LR04), blue bars indicate glacial terminations and younger dryas. (B) Comparison between the ln (Fe/sum) record and the NHSI (LA 2004), green bars indicate periods of high insolation (below).

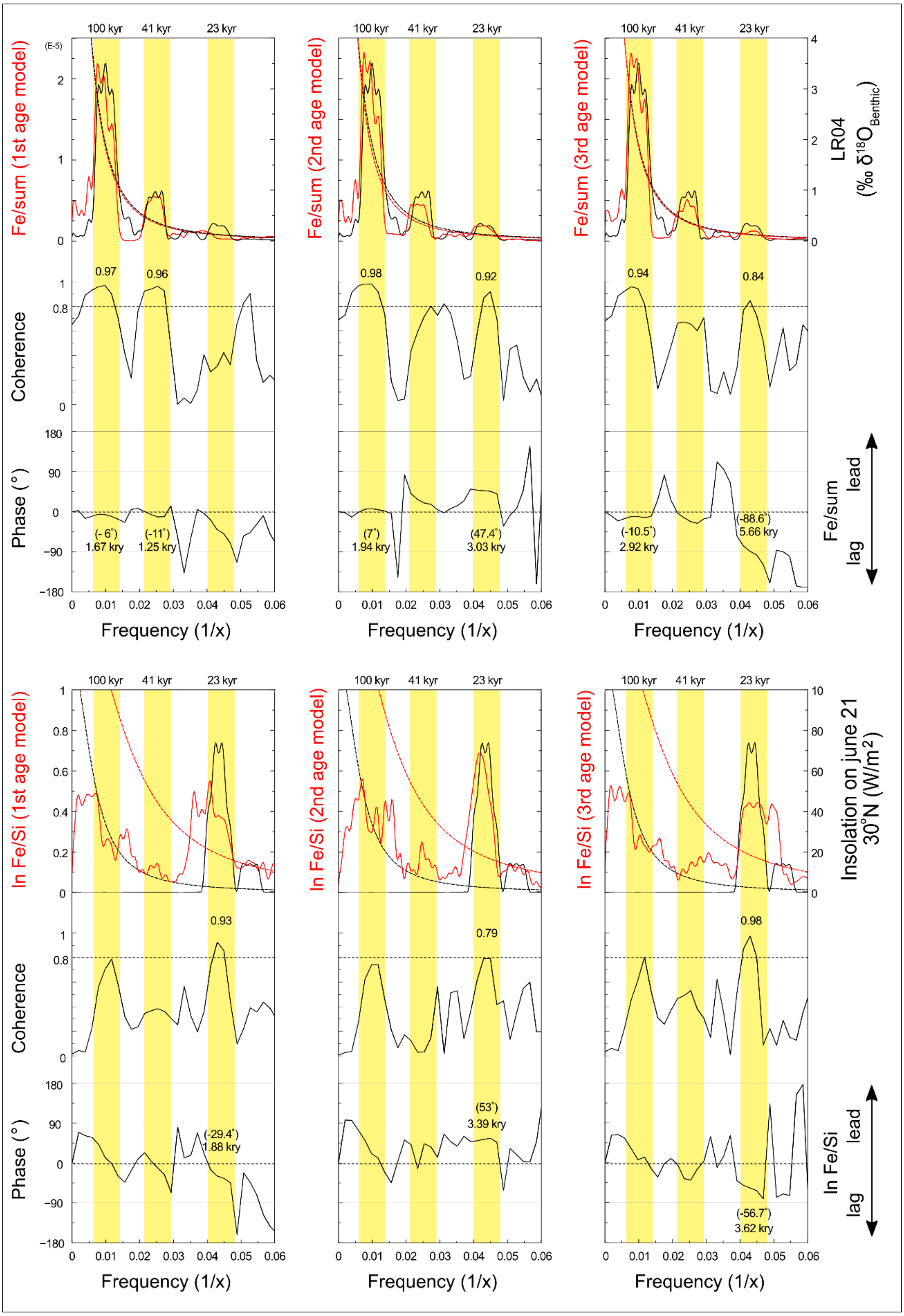

Figure S10. Cross-spectral analyses between continental aridity (Fe/sum) and LR04, and between Indian winter monsoon wind [ln (Fe/Si)] and the NHSI, for the three obtained age models.

Supplementary Tables

1st age model tie points		
Depth (mcd)	Age (Kyr)	sed rate (cm/kyr)
0	0	4.28
0.77	18	2.20
1.74	62	1.57
2.07	83	3.77
2.9	105	5.15
3.93	125	7.87
5.11	140	2.02
6.08	188	6.65
7.21	205	6.78
8.43	223	3.52
9.45	252	3.86
10.8	287	3.61
12.39	331	4.73
12.91	342	3.65
15.17	404	4.47
16.51	434	2.38
17.89	492	3.71
18.67	513	4.44
19.78	538	5.36
20.53	552	

Table S1. Tie points and sedimentation rate obtained by correlating the Sr/sum minima values with LR04 maxima values during glacial maximum and vice-versa.

2nd age model tie points		
Depth (mcd)	Age (Kyr)	sed rate (cm/kyr)
0	0	6.84
0.684444444	10	3.28
1.078636364	22	2.40
1.343181818	33	1.65
1.541590909	45	1.53
1.74	58	0.85
1.85	71	1.29
1.991428571	82	2.86
2.334090909	94	4.31
2.765	104	5.44
3.363500001	115	5.87
4.008666664	126	9.18
5.11	138	2.57
5.392916666	149	1.71
5.615208334	162	1.68
5.817291667	174	1.68
6.019375001	186	5.26
6.545294116	196	4.43
7.077058823	208	4.91
7.616666669	219	6.16
8.294444446	230	4.52
8.746551724	240	2.93
9.098275861	252	4.22
9.604285715	264	3.31
10.06714286	278	3.86
10.53	290	3.76
10.98068182	302	5.78
11.55886364	312	3.94
11.9925	323	3.98
12.39	333	4.33
12.91	345	4.05
13.27451613	354	2.43
13.49322581	363	3.24
13.78483871	372	4.10
14.11290322	380	5.01
14.51387097	388	3.65
14.8783871	398	1.46
15.02419355	408	3.69
15.39333333	418	6.14
15.88466667	426	4.91
16.376	436	4.05
16.70034483	444	2.62
16.96206897	454	2.64
17.2	463	1.30
17.34275862	474	1.90
17.53310345	484	2.16
17.77103448	495	3.42
18.11285714	505	3.04
18.44714286	516	4.04
18.892	527	2.87
19.3804	544	5.09
19.94071429	555	5.81
20.58	566	

Table S2. Tie points and sedimentation rate obtained using the Filtered (Fe/sum) high (low) values, tuned in zero lag phase with the NHSI (74) (June 21st insolation at 30˚N) low (high) values.

3rd age model tie points		
Depth (mcd)	Age (Kyr)	sed rate (cm/kyr)
0	0	2.581
0.8	31	2.879
2.49	89.7	4.542
3.73	117	7.905
4.56	127.5	3.942
5.1	141.2	2.559
6.075	179.3	3.958
7.195	207.6	8.248
8.457	222.9	3.653
9.447	250	3.807
10.277	271.8	3.939
10.797	285	4.576
11.186	293.5	3.550
12.276	324.2	4.643
13.056	341	2.800
14.456	391	4.067
15.066	406	3.937
15.696	422	6.000
16.356	433	2.239
17.469	482.7	5.342
17.859	490	4.050
18.669	510	

Table S3. Tie points and sedimentation rate obtained by correlating the U1471 Sr/sum record, with the U1467 Sr/sum record (75).

References

56. Paul, A., Reijmer, J., Lampart, J., Kinkel, H. & Betzler, C. Relationship between Late Pleistocene sea-level variations, carbonate platform morphology and aragonite production (Maldives, Indian Ocean). *Sedimentology* **59**, 1640–1658 (2012).
57. Betzler, C. *et al.* Site U1471. in (2017). doi:10.14379/iodp.proc.359.109.2017.
58. Droxler, A. Pliocene-Pleistocene Aragonite Cyclic Variations in Holes 714A and 716B (the Maldives) Compared with Hole 633A (the Bahamas): Records of Climate-Induced CaCO3 Preservation at Intermediate Water Depths. *Proc. Ocean Drill. Program* (1990).
59. Betzler, C. *et al.* Monsoon-induced partial carbonate platform drowning (Maldives, Indian Ocean). *Geology* **37**, 867–870 (2009).
60. Aerosol Optical Depth. https://earthobservatory.nasa.gov/global-maps/MODAL2_M_AER_OD (2022).
61. Andreae, M. O. & Gelencsér, A. Black carbon or brown carbon? The nature of light-absorbing carbonaceous aerosols. *Atmospheric Chem. Phys.* **6**, 3131–3148 (2006).
62. Laskin, A., Laskin, J. & Nizkorodov, S. A. Chemistry of Atmospheric Brown Carbon. *Chem. Rev.* **115**, 4335–4382 (2015).
63. Moosmüller, H., Chakrabarty, R. K. & Arnott, W. P. Aerosol light absorption and its measurement: A review. *J. Quant. Spectrosc. Radiat. Transf.* **110**, 844–878 (2009).
64. Russell, P. B. *et al.* Absorption Angstrom Exponent in AERONET and related data as an indicator of aerosol composition. *Atmospheric Chem. Phys.* **10**, 1155–1169 (2010).
65. Ackerman, S. A. & Cox, S. K. Surface weather observations of atmospheric dust over the southwest summer monsoon region. *Meteorol. Atmospheric Phys.* **41**, 19–34 (1989).
66. Nair, R. R. *et al.* Increased particle flux to the deep ocean related to monsoons. *Nature* **338**, 749–751 (1989).
67. Yu, Y., Notaro, M., Kalashnikova, O. V. & Garay, M. J. Climatology of summer Shamal wind in the Middle East. *J. Geophys. Res. Atmospheres* **121**, 289–305 (2016).
68. L3 Browser - NASA Ocean Color. https://oceancolor.gsfc.nasa.gov/l3/.
69. Kunkelova, T. *et al.* A two million year record of low-latitude aridity linked to continental weathering from the Maldives. *Prog. Earth Planet. Sci.* **5**, 86 (2018).
70. Lindhorst, S., Betzler, C. & Kroon, D. Wind variability over the northern Indian Ocean during the past 4 million years – insights from coarse aeolian dust (IODP Exp. 359, Site U1467, Maldives). *Palaeogeogr. Palaeoclimatol. Palaeoecol.* **536**, 109371 (2019).
71. Murty, A. Monsoons over the Indian Subcontinent. *Acad. Lett.* (2021).
72. Bunzel, D. *et al.* A multi-proxy analysis of Late Quaternary ocean and climate variability for the Maldives, Inner Sea. *Clim. Past* **13**, 1791–1813 (2017).
73. Lisiecki, L. E. & Raymo, M. E. A Pliocene-Pleistocene stack of 57 globally distributed benthic δ18O records. *Paleoceanography* **20**, (2005).
74. Laskar, J. *et al.* A long-term numerical solution for the insolation quantities of the Earth. *Astron. Astrophys.* **428**, 261–285 (2004).
75. Stainbank, S. *et al.* Long-term, high-resolution foraminiferal geochemical records ($\delta^{18}O$, $\delta^{13}C$) from IODP Site 359-U1467. (2020) doi:10.1594/PANGAEA.914883.
